# Immune checkpoints in immune response to glioma: two sides of the same coin

**DOI:** 10.3389/fimmu.2025.1639521

**Published:** 2025-08-15

**Authors:** Oxana Musatova, Vikas Kumar, Konstantin Vinogradov, Yury Rubtsov

**Affiliations:** ^1^ Shemyakin-Ovchinnikov Institute of Bioorganic Chemistry, Russian Academy of Science, Moscow, Russia; ^2^ Department of Bioinformatics and Systems Biology, Phystech School of Biological and Medical Physics, Moscow Institute of Physics and Technology, Dolgoprudny, Moscow Region, Russia

**Keywords:** glioma, glioblastoma, immune response, immune checkpoints, T cell exhaustion, myeloid suppressor cells, regulatory T cells, immune checkpoint inhibitors

## Abstract

Gliomas are aggressive brain tumors of glial origin accounting for about 80% of the central nervous system (CNS) malignancies. Glioma cells are known to form a highly immunosuppressive tumor microenvironment (TME) capable of inhibiting T cell activation and protecting tumors from elimination by the immune system. One of the predominant immune inhibitory mechanisms in the TME are immune checkpoints: a complex system of membrane-bound ligands on tumor and immune cells that interact with surface receptors on T lymphocytes and affect their activation and cytotoxicity. There is mounting evidence regarding the role of immune checkpoints expressed in gliomas, in particular, their most aggressive form – glioblastoma multiforme (GBM). In this review, we discuss the immune checkpoints with proven expression in gliomas, their ligands, related signaling pathways, co-expression profiles, and the effects of immune cells on antitumor activity. We collected data not only on the canonical immune checkpoints (e.g. PD-1/PD-L1 or CTLA-4) but also on novel and alternative ones including soluble mediators and enzymes. We review data describing the correlation of immune checkpoint expression with patient survival as well as co-expression with other molecules involved in glioma development. Where possible, we analyzed the differences between immune checkpoints in low-grade (LGG) and high-grade gliomas (HGG). Negative effects of several immune checkpoints on T cells could be eliminated by therapeutic monoclonal antibodies that block the interaction between checkpoint ligands and receptors. Therefore, alongside with traditional approaches and T cell-based immunotherapy, the antibody-mediated blockade of immune checkpoints could be considered as a potentially promising therapeutic approach against gliomas.

## 1 Introduction

Glioma is the most common type of primary CNS tumor. Gliomas are classified into four grades based on both histological features and molecular markers (1). Grade I and II gliomas are defined as low-grade gliomas (LGG), while high-grade gliomas (HGG) include grade III and IV gliomas. LGG patients show a better prognosis and survival (up to 13 years) (2). However, LGGs often develop into HGG (3). The 2-year survival rate of HGG patients does not exceed 20% (4). Grade IV glioma is usually called glioblastoma (GBM) and is characterized by its aggressiveness, therapy resistance, and a very high risk of relapse (5). The 5-year survival rate for GBM patients is only 5.6% (1). The incidence of glioma and GBM is estimated at 5.89 and 3.26 cases per 100 000 people, respectively, depending on gender, age, and race (1).

The established gold standard of treatment for patients with new cases of GBM is known as the Stupp protocol and includes surgical resection, radiation therapy, and chemotherapy with the alkylating agent temozolomide (TMZ) (6). The treatment of GBM begins with a maximal surgical resection that removes the majority of tumor cells and provides a material for proper histologic diagnosis and molecular testing. Surgical resection is followed by six weeks of radiation therapy (60 Gray [Gy] in 2-Gy fractions) and concomitant daily TMZ (75 mg/m2), followed by six cycles of adjuvant TMZ (150–200 mg/m2). The Stupp protocol has remained unchanged for the past 18 years and typically provides patients with an overall survival of less than two years. Despite these first-line treatments, GBM almost always recurs (5).

GBM resistance to therapy and almost inevitable relapses can be explained by its specific anatomic location (CNS) and high invasive potential which makes its complete surgical resection almost impossible (7). The blood-brain barrier is a hurdle for GBM drug therapy with chemotherapeutics or monoclonal antibodies (8). High heterogeneity of tumors from different patients and of GBM cells within a single particular tumor makes the development of efficient target drugs against GBM a compelling challenge (9).

Gliomas have been shown to possess a well-developed immunosuppressive molecular machinery (reviewed in 10). They are prone to infiltration by immune cells but, contrary to expectations, this has an opposite effect promoting tumor progression. The most viable explanation lies in the nature of GBM-infiltrating cells, such as various macrophage subsets. Gliomas have been demonstrated to release a set of molecules that modulate immune responses (11). GBM secretes extracellular vesicles and factors, such as ARG1 or TGF-β, which recruit macrophages and switch the polarization to protumor M2 phenotype, forming tumor-infiltrating macrophages (TAM) (11). The enhanced regulatory T cells (Treg) infiltration and expansion in TME was also detected. In contrast, effector cell infiltration is remarkably reduced. The stimulation of the immunosuppressive populations of immune cells, at the same time, inhibits, exhausts and promotes apoptosis of tumor-reactive immune cells (11). In this review, we briefly describe immune checkpoint molecules found in glioma cells, glioma microenvironment or the in-patients’ biological fluids. We focus on the role of each immune checkpoint molecule in glioma growth and immune escape. In addition, we discuss the evidence in favor of the impact of immune checkpoint expression levels on glioma patients’ survival, both in case of LGG and HGG, where possible. Therefore, we would like to emphasize that therapeutic approaches targeting the immune checkpoints have to be carefully evaluated to avoid any potential complications before transition from bench to bedside.

## 2 Immunoglobulin superfamily immune checkpoints

### 2.1 PD-1 pathway

**Figure 1:**
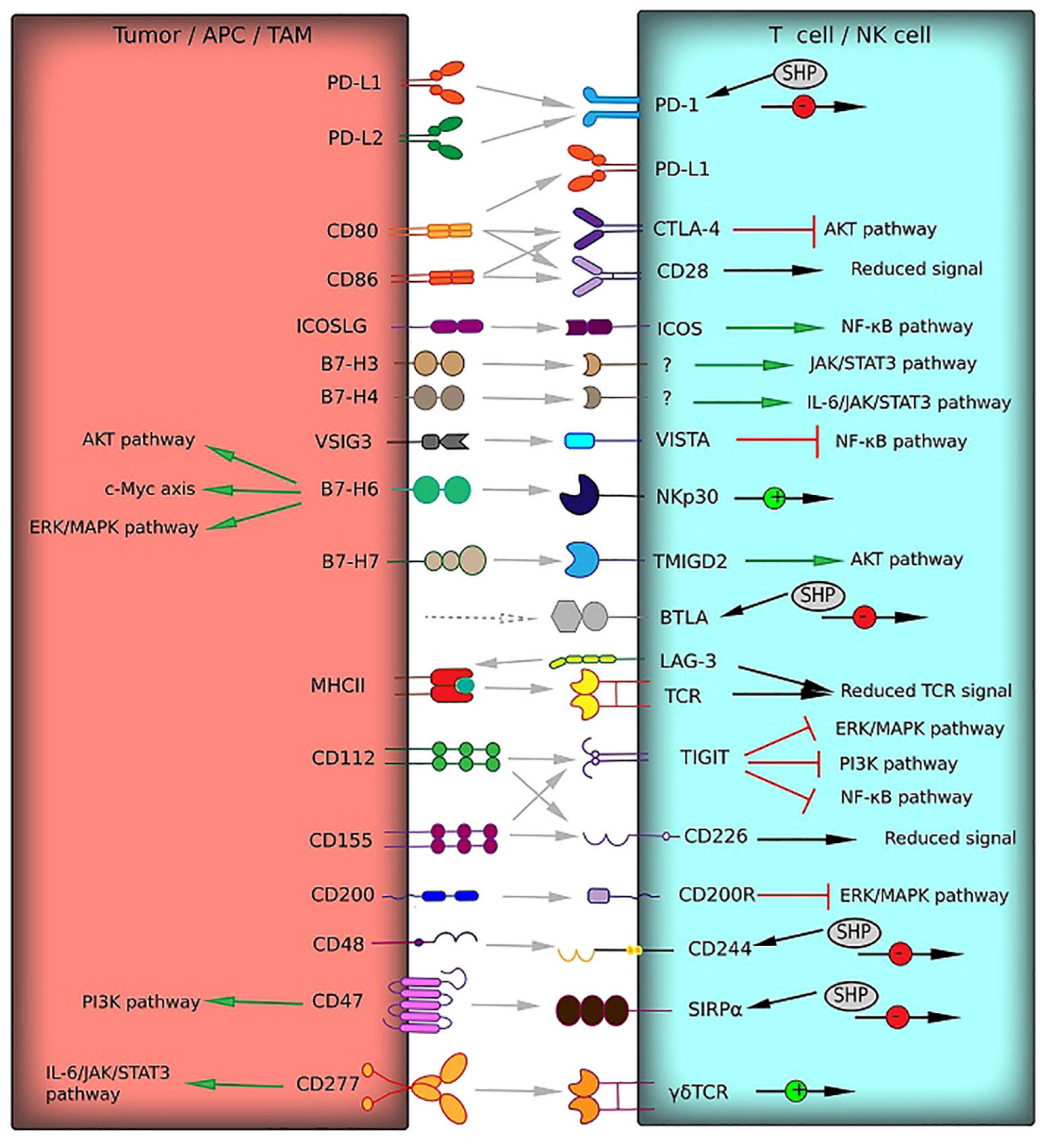
Receptor–ligand interactions (gray arrows) of immune checkpoints from the immunoglobulin superfamily between T cells and tumor cells or TAM can mediate T cell responses. These interactions can activate co-stimulatory signals (green arrows) or deliver inhibitory signals (red square arrows). The signaling pathways involved in activation or inhibition are indicated. Multiple effects on signaling pathways are shown by black arrows with green (activation) or red (inhibition) circles. HVEM and Gal-3, which interact with BTLA and LAG-3, respectively (gray dotted arrows), belong to other protein families and are not presented in this figure.

Immunoglobulin superfamily surface molecules are implicated in the propagation of the stimulatory and inhibitory signals in the immune cell lineages (**Figure 1**). The most widespread and thoroughly described immune checkpoint involves the Programmed cell death 1 (PD-1) and its ligand, Programmed cell death 1 ligand 1 (PD-L1, B7-H1). The interaction between PD-1 on the T cell surface and its ligand mediates multiple immunosuppressive effects such as apoptosis and functional exhaustion of conventional T cells (Tconv), reduced cytokine secretion, and generation of Tregs and TAMs (12). PD-L1 is expressed in numerous neoplasms, including brain tumors. PD-L1 overexpression was observed in about 90% of GBM tumor cells and GBM-associated macrophages (13). Moreover, GBM-infiltrating CD4^+^ and CD8^+^ lymphocytes have been shown to express both PD-1 and PD-L1, which indicates induction of Tregs and reprogramming of Tconv to the self-inhibiting lymphocytes (14). Tumor PD-L1 can be exposed on the surface of GBM-derived extracellular vesicles and delivered to the distant sites by bloodstream (15). The cytoplasmic region of PD-1 contains the immunoreceptor tyrosine-based switch motif (ITSM), which recruits Src homology region 2 domain-containing phosphatase-2 (SHP-2). Recruited SHP-2 mediates dephosphorylation of TCR-associated CD3 and ZAP70 and inhibits CD28 co-stimulatory signals. It leads to the deactivation of pathways such as PI3K/Akt and NF-κB, which results in reduced transcriptional activity, inactivation of the pathways downstream of the T-cell receptor (TCR) and lower IL-2 production (16). Another mechanism of PD-1/PD-L1 axis involves shielding the costimulatory molecules CD80 by PD-L1. PD-L1 has been shown to interact with CD80, thereby preventing its binding to CD28, which is required for the stimulation of T cells by antigen-presenting cells (APCs) (17).

The effects of PD-L2 (CD273), the second PD-1 ligand, on the immune activation are similar to PD-L1, but still not the same. However, unlike PD-L1, PD-L2 has only one receptor – PD-1. PD-L2 is not as widespread in tumors, which indicates its secondary role in forming the inhibitory TME (18). Nevertheless, PD-L2 overexpression has been detected in HGG and is associated with the wild-type status of isocitrate dehydrogenase 1 (IDHwt) and a highly invasive mesenchymal GMB phenotype. To sum up, overexpression of PD-1 and both ligands correlate with a large count of GBM-induced Tregs and a bad prognosis for patients (19–22).

### 2.2 CTLA-4

Cytotoxic T-lymphocyte associated protein 4 (CTLA-4) plays a key role in tumor immune reactions with PD-1. CTLA-4 is homologous to the costimulatory T cell receptor CD28 and binds to the same ligands CD80 and CD86, but with a significantly higher affinity (23). Thus, tumor-infiltrating lymphocytes (TILs) express CTLA-4 to disrupt the costimulatory signaling by shielding CD80 and CD86 from CD28 in a manner similar to PD-L1 (23). At a molecular level, CTLA-4 signaling inhibits AKT phosphorylation and activation of the transcription factors, such as NF-κB, AP-1, and NF-AT, induced by co-stimulatory CD28 (24). Blocking CTLA-4 by monoclonal antibodies protects T cells from negative regulation and restores antitumor immune reactions. This makes CTLA-4 inhibitors promising antitumor agents, since CTLA-4 is involved in cancer development, including brain tumors (**Table 1**) (35). Elevated CTLA-4 expression was detected in HGG patients mostly characterized by the IDHwt status and mesenchymal cell type. CTLA-4 overexpression leads to a lower survival of HGG and LGG patients. There is a strong correlation of CTLA-4 levels and enhanced tumor infiltration with Treg and inhibitory macrophages. CTLA-4 expression also correlates with the expression of other immune checkpoints such as PD-1, CD40, ICOS, and TIGIT (36).

**Table 1:** Clinical trials of immune checkpoint inhibitors for glioma therapy.

Name	Target	Examples of clinical trials	Tumor description	Median overall survival (mOS), months	Comments	References
Nivolumab	PD-1	NCT02667587: An Investigational Immunotherapy Study of Temozolomide Plus Radiation Therapy With Nivolumab or Placebo, for Newly Diagnosed Patients With Glioblastoma (GBM, a Malignant Brain Cancer)	Primary GBM, MGMT-Methylated promoter	28.9	mOS was 32.1 months in placebo group; did not improve survival	(25)
		NCT02617589: An Investigational Immunotherapy Study of Nivolumab Compared to Temozolomide, Each Given with Radiation Therapy, for Newly-diagnosed Patients with Glioblastoma (GBM, a Malignant Brain Cancer) (CheckMate 498)	Primary GBM, unmethylated MGMT promoter	13.4	TMZ + RT demonstrated a longer mOS (14.9 months)	(26)
		NCT02017717: A Study of the Effectiveness and Safety of Nivolumab Compared to Bevacizumab and of Nivolumab With or Without Ipilimumab in Glioblastoma Patients (CheckMate 143)	First diagnosis of unmethylated MGMT GBM or first recurrence of GBM	9.8	mOS was 10.0 months for bevacizumab (anti-VEGF) control group	(27)
Pembrolizumab	PD-1	NCT02054806: A Study of Pembrolizumab (MK-3475) in Participants With Advanced Solid Tumors (MK-3475-028/KEYNOTE-28)	Recurrent PD-L1-positive GBM	13.1	–	(28)
		NCT02337491: Pembrolizumab +/- Bevacizumab for Recurrent GBM	First or second relapse of GBM or gliosarcoma if the original tumor histology was LGG or GBM	10.3	mOS was 8.8 months for combined therapy	(29)
Durvalumab	PD-L1	NCT02336165: Phase 2 Study of Durvalumab (MEDI4736) in Patients With Glioblastoma	Primary GBM with unmethylated MGMT promoter, first or second recurrence of GBM	15.1	20% patients remained alive with ongoing survival ranging from 15.7 to 34.9 months	(30)
Avelumab	PD-L1	NCT03047473: Avelumab in Patients With Newly Diagnosed Glioblastoma Multiforme (SEJ)	Newly diagnosed GBM or lower grade astrocytoma that has been upgraded to a histologically verified GBM	15.3	There was no apparent improvement in survival in comparison with Stupp protocol (15 months)	(31)
Ipilimumab	CTLA-4	NCT03367715: Nivolumab, Ipilimumab, and Short-course Radiotherapy in Adults With Newly Diagnosed, MGMT Unmethylated Glioblastoma	Newly Diagnosed MGMT Unmethylated Glioblastoma	16.85	–	(32)
		NCT02311920: Ipilimumab and/or Nivolumab in Combination With Temozolomide in Treating Patients With Newly Diagnosed Glioblastoma or Gliosarcoma	Newly diagnosed GBM after resection and chemoradiation	20.7	–	(33)
Tremelimumab	CTLA-4	NCT02794883: Tremelimumab and Durvalumab in Combination or Alone in Treating Patients With Recurrent Malignant Glioma	Grade III or IV glioma	7.246 (Tremelimumab) 11.71 (Durvalumab) 7.703 (Mix)	–	(32)
BMS-986016	LAG-3	NCT02658981: Anti-LAG-3 Alone & in Combination w/Nivolumab Treating Patients w/Recurrent GBM	Primary progressive or recurrent GBM or gliosarcoma	8	3 out of 16 patients in the combination therapy lived beyond 20 months at the end of phase I	(34)

### 2.3 ICOS/ICOSLG

ICOSLG (B7-H2, or CD275), the ligand of the inducible T cell costimulatory protein (ICOS), is expressed on the surface of somatic cells and APCs. Despite the positive costimulatory role of the ligand, the outcome of ICOS/ICOSLG binding depends on TME. During tumor development, ICOSLG can both promote and suppress tumor progression, since it activates Tconv cells and, at the same time, induces Tregs through NF-κB signaling (37). The TME is characterized by increased expansion and infiltration of Tregs and suppression of Teff functions (11), therefore, ICOSLG has a greater effect on Treg. ICOSLG is expressed on GBM tumor cells, its upregulation being associated with the presence of glioblastoma stem cells and IL-10-producing T cells as well as the mesenchymal phenotype. As a result, patients with ICOSLG overexpression have a lower overall survival (37). These data are in line with evidence that ICOS is overexpressed in glioma-infiltrating Tregs, as well as with the established link between high ICOS levels and a bad prognosis (38). Nevertheless, protein distribution in the body is also important. For instance, low ICOS levels in the blood plasma of GBM patients was associated with a global immunosuppression and the lower overall survival (39). ICOS/ICOSLG was shown to be co-expressed with a number of inhibitory immune checkpoints such as PD-1/PD-L1/PD-L2 (38).

### 2.4 B7-H3

B7 homolog 3 (B7-H3, CD276) is a type I transmembrane protein which exerts immunosuppressive activity by triggering T cell exhaustion. In healthy tissues, B7-H3 can be found on resting fibroblasts and osteoclasts, endothelial cells, activated T cells, natural killer cells (NK) and APCs. The inhibitory function of B7-H3 is widely used by tumors, including gliomas. Patients with IDHwt HGG have high levels of B7-H3 (40, 41). B7-H3 overexpression also correlates with a lower survival in LGG patients (42). Duerinck et al. studied the mutually exclusive expression profiles of B7-H3 and PD-L1 and suggested B7-H3 to be the major factor responsible for the failure of anti-PD-1 and anti-CTLA-4 HGG therapy (35).

The B7-H3 signaling cascade involves the activation of JAK2/STAT3 survival pathway leading to tumor growth and epithelial–mesenchymal transition in glioma cells. The exosomal transport of B7-H3 can also enhance tumor aggressiveness and facilitate immune escape in medulloblastoma (43) and neuroblastoma (44). Nevertheless, the role of B7-H3 in the immune response against GBM remains controversial. The receptor for B7-H3 has not been identified yet, but it is mostly likely present on the surface of activated CD4^+^ and CD8^+^ cells (45, 46). B7-H3 can have several candidate receptors, since B7-H3 was shown to act not only as an inhibitory molecule, but also as a stimulatory one (47).

### 2.5 B7-H4

B7 homolog 4 (B7-H4, VTCN1, B7x, B7S1) is a type I transmembrane protein of B7 family. Normally, B7-H4 is expressed by dendritic cells (DCs) and APCs. Although the B7-H4 overexpression was detected in several types of cancer, it is not considered as a typical tumor marker (48). B7-H4 levels have been shown to correlate positively with a tumor grade and a poor prognosis in glioma patients (49). B7-H4 production in GBMs has been shown to depend on IL-6 signaling via IL-6/JAK/STAT pathway activation and is associated with an elevated number of TAMs (50). B7-H4 levels does not correlate with expression of other immune checkpoints. For instance, B7-H4 and B7-H3 co-expression was observed only in 10% of GBM cases. Interestingly, B7-H4 and PD-L1 were co-expressed only in 2% of gliomas, making it most reasonable to assume a possible functional redundancy of these molecules (51). Similar, to B7-H3, the receptor for B7-H4 is still unknown.

### 2.6 VISTA

VISTA (V-domain Ig suppressor of T cell activation), also known as B7-H5, is highly expressed in myeloid cells and TILs. VISTA acts as an activating ligand for APCs and an inactivating one for T cells (41). VISTA suppresses T cell proliferation and cytokine production by inhibiting NF-κB pathway (52). VISTA is commonly expressed in tumors and positively correlates with the WHO tumor grade and a poor prognosis for glioma patients (53). Moreover, VISTA is often co-expressed along with other inhibitor immune checkpoints such as B7-H3, PD-1, PD-L1, LAG-3, TIM-3 (54).

To date, two receptors for VISTA have been identified. VSIG-3 (IgSF11) is a member of the immunoglobulin superfamily which is highly expressed in gliomas. VSIG-3 is usually associated with high-grade malignancies and a worse outcome (55). The interaction between VSIG-3 and VISTA inhibits T cell proliferation and production of proinflammatory cytokines and chemokines (56).

PSGL1 (selectin P ligand), also known as SELPLG or CD162, is another receptor for VISTA. It has been suggested that PSGL1 stimulation may inhibit AKT and ERK signaling induced by TCR stimulation in some tumors. PSGL1 was detected on HGG and is co-expressed with VISTA (55). However, PSGL1 has not been studied thoroughly in the context of brain tumors.

### 2.7 B7-H6

B7 homolog 6 (B7-H6), or NCR3LG1, is a B7 family immune checkpoint protein which acts as an endogenous costimulatory ligand. The extracellular domain of NKp30 on the surface of NK cells is a receptor for the extracellular part of B7-H6 (57). B7-H6 binding to NKp30 induces NK cells activation. This protein is almost absent in normal tissues and mononuclear cells from the peripheral blood of healthy donors; however, it can be detected on the surface of neutrophils and proinflammatory macrophages in the presence of proinflammatory cytokines such as TNF- α, IL-1β, or TLR ligands (58). B7-H6 is also selectively expressed on a range of brain tumor cells such as human neuroblastoma (59), astrocytoma (60), and glioma (61). B7-H6 overproduction positively correlates with tumor aggressiveness and a poor prognosis. In gliomas, B7-H6 regulates a spectrum of biological processes such as proliferation, migration, invasion, survival, and cell cycle control by activating the PI3K/Akt, ERK/MAPK, and c-Myc/RNMT signaling pathways (62).

### 2.8 B7-H7

B7 homolog 7, also known as HHLA2, is not expressed in healthy tissues, except for the placenta, gut, kidney, breast tissues, and macrophages. HHLA2 is absent in the brain, even in glial cells and neurons; however, it was detected in endothelial cells. HHLA2 is highly expressed in tumors, and at low levels, it was found in LGGs and, less frequently, in HGGs (63). HHLA2 expression is downregulated with tumor progression. Moreover, HHLA2 overexpression is associated with the prolonged overall survival in GBM patients (63). HHLA2 was shown to interact with CD28H and stimulate T cell proliferation and cytokine production via AKT phosphorylation (64). Nevertheless, there is evidence indicating that high B7-H7 expression in other cancer types is associated with a poor prognosis. For example, HHLA2 was found to be highly expressed in osteosarcoma and colorectal carcinomas and positively correlated with metastasis and a poor prognosis (65, 66). It is assumed that HHLA2 has at least two ligands with opposing functions, making it in a way similar to B7-H3. CD28H or TMIGD2 is the confirmed HHLA2 ligand with stimulatory activity, while the second ligand with an inhibitory activity has not been identified yet (67).

### 2.9 LAG-3

LAG-3 (Lymphocyte-activation Gene-3, or CD223) is expressed on microglial cells (68). LAG-3 is closely related to CD4 and can bind to MHC II (69). LAG-3 triggers CD4^+^ T cell exhaustion and limits T cell proliferation by competing for Zn ions with Lck causing its dissociation from complex with TCR. Cleavable by ADAM10/17 proteases cytoplasmic C-terminus of LAG-3 contains domains rich in glutamic acid which are responsible for acidification and withdrawal of Zn. LAG-3 presence in TME was associated with an enhanced CD8^+^ T cells infiltration, PD-1^+^ TILs and PD-L1^+^ IDHwt glioma cells (70). LAG-3 overexpression correlated with a poor prognosis in LGG patients (71). However, the role of LAG-3 in HGG remains controversial. TILs in GBM TME were shown to express higher LAG-3 levels compared to lymphocytes from healthy donors (72). LAG-3 co-expression with CTLA-4, PD-1, and TIM-3 (73) is considered to be a risk factor in GBM patients based on bioinformatics studies (74). The role of LAG-3 alone on survival and prognosis in GBM patients is not clear.

The first described LAG-3 ligand is galectin-3 (Gal-3), a β-galactose-binding lectin involved in proliferation, cell adhesion, and apoptosis. Although galectin-3 is a proven immunomodulator, it is also considered as a glioma-related marker. Gal-3 expression was reported to correlate with the WHO grade of gliomas (75). Fibrinogen-like protein 1 (FGL1) is another functional LAG-3 ligand. Soluble FGL1 from the blood stream induces the surface LAG-3 and transmits an inhibiting signal to T cells (76). However, the role of FGL1 in glioma development is very complex and poorly understood (77).

### 2.10 CD155/CD112 pathways

CD155, also known as the poliovirus receptor (PVR), is a glycoprotein which belongs to the immunoglobulin superfamily. Its expression is inherent in malignant cells and is rarely found in normal tissues, except epithelial or endothelial cells. HGGs, including GBMs, are typically associated with CD155 overexpression (78) and with a lower survival rate. The same tendency was detected for LGG (79). CD155 has emerged as a tumor promoting antigen, upregulated on GBM and related to increased GBM aggressiveness and metastasis (80). The functions of this receptor were shown to depend on engaging ligands. It can activate NK cells by binding CD226 (T lineage specific activation antigen 1, TLisA1) and CD96 (Tactile) and, on the contrary, inhibit them by triggering TIGIT (81). In GBM, CD155 promotes TIGIT^+^ immune cell infiltration and the transition of the circulating NK cells to TIGIT^+^/CD226^-^ phenotype, while normally TIGIT^+^ NK cells are absent in the CNS and peripheral blood (82).

TIGIT is a co-inhibitory receptor which could bind both CD155 (with high affinity) and CD112 (nectin-2, with low affinity) (83). It is expressed on the surface of immune cells such as memory and activated T cells, Tregs, NK, and NKT cells. TIGIT binds CD155 with higher affinity compared to CD226, preventing NK cell stimulation and function via the CD155/CD226 pathway (84). CD112 is another co-inhibitory NK cell receptor, and its binding to TIGIT also contributes to inhibiting NK cells. This prevents IFN-γ secretion and cytolytic granule release by NK-cells (85). TIGIT is overexpressed in GBM TILs and peripheral blood T cells of patients with GBM as compared to lymphocytes from healthy donors. Nevertheless, in most patients, co-stimulating factor CD226 was also overexpressed in GBM-infiltrating immune cells along with TIGIT (82). It implies the possible competition for the ligand with prevalent CD155/TIGIT binding and subsequent inhibition of NK cell function (82). The co-expression of CD155 and PD-L1 was confirmed for tumor cells and TAMs (85), while TIGIT and PD-1 were upregulated on TILs and associated with poor overall survival (82, 86).

### 2.11 CD200

CD200, another member of the immunoglobulin superfamily, has recently been recognized as an immune checkpoint. This protein is expressed on various immune and stromal cells as well as tumor cells. In gliomas, CD200 facilitates tumor growth both *in vivo* and *in vitro.* Furthermore, the soluble form of this protein is carried to the cervical lymph nodes through the cerebral spinal fluid contributing to the suppression of lymphocytes (87). Soluble CD200 in the patient’s bloodstream contributes to systemic immunosuppression. The main mechanism of CD200-mediated immunosuppression is likely to be the switching of macrophage polarization toward the M2 phenotype and inducing myeloid-derived suppressor cells (MDSC) in TME (88). In GBM patients, a high plasma level of CD200 was associated with an increased accumulation of MDSCs (88). The role of this protein in GBM development is actively investigated, and to date, there are no studies reporting the link between CD200 expression and the prognosis in patients with brain tumors, further studies are required for better understanding of diagnostic/therapeutic potential of this molecule.

### 2.12 CD48

CD48 is an immune checkpoint, also known as the B-lymphocyte activation marker (BLAST-1) or signaling lymphocytic activation molecule 2 (SLAMF2). CD48 is expressed on cells of hematopoietic origin, especially on APCs. CD48 is a key molecule in immunological synapses and is essential for co-stimulation. It binds to CD2 and promotes T cell activation, as well as the function of granulocytes and NK cells (89). Despite the ability to activate immune cells, CD48 binding with the high-affinity receptor 2B4 (CD244, SLAMF4) results in NK cell dysfunction. CD48 expression was shown for several oncologic pathologies, particularly glioblastoma (90). CD48 upregulation in gliomas was associated with enhanced macrophage and T cell infiltration, the IDHwt status of mesenchymal subtype gliomas and a worse outcome. CD48 has a strong association with most checkpoints such as TIGIT, ICOS, TIM-3, but not with CTLA-4 and PD-L1 (91).

### 2.13 CD47

CD47, also known as integrin-associated protein (IAP), is a transmembrane protein from the immunoglobulin superfamily. Normally, CD47 regulates phagocytosis through the interaction with SIRP-α receptors on macrophages (92). Several malignancies including gliomas express CD47. CD47 signaling was associated with AKT phosphorylation and PI3K/Akt pathway which resulted in tumor maintenance and survival (93). CD47 was associated with glioma stem-like cells and predicts a worse prognosis for patients (94).

### 2.14 CD277

CD277 or BTN3A1 (Butyrophilin subfamily 3 member A1) is a member of the immunoglobulin superfamily typically expressed on T cells, B cells, NK, DCs, and tumor cells (95). The role of CD277 in tumor progression is still poorly understood, but it can bind particular variants of γδ TCR causing their activation and cytotoxicity. There is a study indicating a carcinogenic role of CD277 in gliomas. According to (96), IDHwt glioblastomas expressed higher CD277 levels compared to WHO grade II and III astrocytomas and oligodendrogliomas. CD277 upregulation was associated with multiple effects on immune system including increased macrophage, B cell, and T cell infiltration as well as CD8^+^ T cell exhaustion (96). Moreover, CD277 was co-expressed with TIM-3, IL-10, and FoxP3 which correlated with a poor prognosis. CD277-dependent activation of the IL-6/JAK/STAT3 pathway may explain its co-expression with TIM-3, which activates the same pathway in cancer cells (96). However, unexpectedly, CD277 in GBMs was reported to trigger the anti-tumor immune responses in γδ T cells (97).

## 3 TNF – TNFR superfamily

### 3.1 HVEM and HVEM-mediated signaling

HVEM, or TNFRSF14, belongs to the tumor necrosis factor receptor (TNFR) family. HVEM is expressed on epithelial and mesenchymal cells and on resting lymphocytes, Tregs, NK and myeloid cells. HVEM was shown to activate as well as inactivate immune responses depending on the ligand (98). HVEM has several ligands: BTLA, CD160, gD, LIGHT, and LTα3 (98). The interaction between HVEM and BTLA or CD160 inhibits T cell functions, whereas its binding to LTα or LIGHT results in T cell activation (99). The expression of HVEM, LIGHT and BTLA was detected in gliomas (100). Aggressive subtypes of gliomas were shown to upregulate HVEM. Using immunohistochemistry, HVEM in gliomas was found predominantly in the microvascular proliferation region and at the edges of the necrotic zone (100). High HVEM levels predict a poor outcome (100). HVEM^high^ GBM tumors tend to contain larger numbers of immune and stromal cells in glioma microenvironment compared to the tumors with a low HVEM level. In glioblastoma samples, HVEM expression was shown to coincide with TIM-3, PD-1, PD-L1, CTLA-4, LAG-3, and VISTA (100).

HVEM is the only reliably identified BTLA receptor. BTLA (also known as CD272) is a transmembrane glycoprotein and the main inhibitory receptor on T cells. BTLA is found on the surface of immune cells, such as B and T lymphocytes, NK and NKT cells, myeloid cells. The interaction between BTLA and HVEM induces a branching signal, which promotes a proinflammatory signal by activating NF-κB (100), and, simultaneously, passes an inhibitory signal by recruiting tyrosine phosphatases SHP-1 or SHP-2, similarly to PD-1 (101). BTLA and HVEM molecules interact when expressed in cis on the membrane of the same cell or in trans on different cells (102). Interestingly, upon the cis-interaction of BTLA and HVEM, the inhibitory function of BTLA prevails over the activating function of HVEM (102). Moreover, BTLA/HVEM cis-binding shields HVEM molecules from BTLA molecules in trans as well as from the stimulation by other activating ligands such as LIGHT (103). Currently, the role of BTLA in glioma development is not well studied and requires future research.

**Figure 2:**
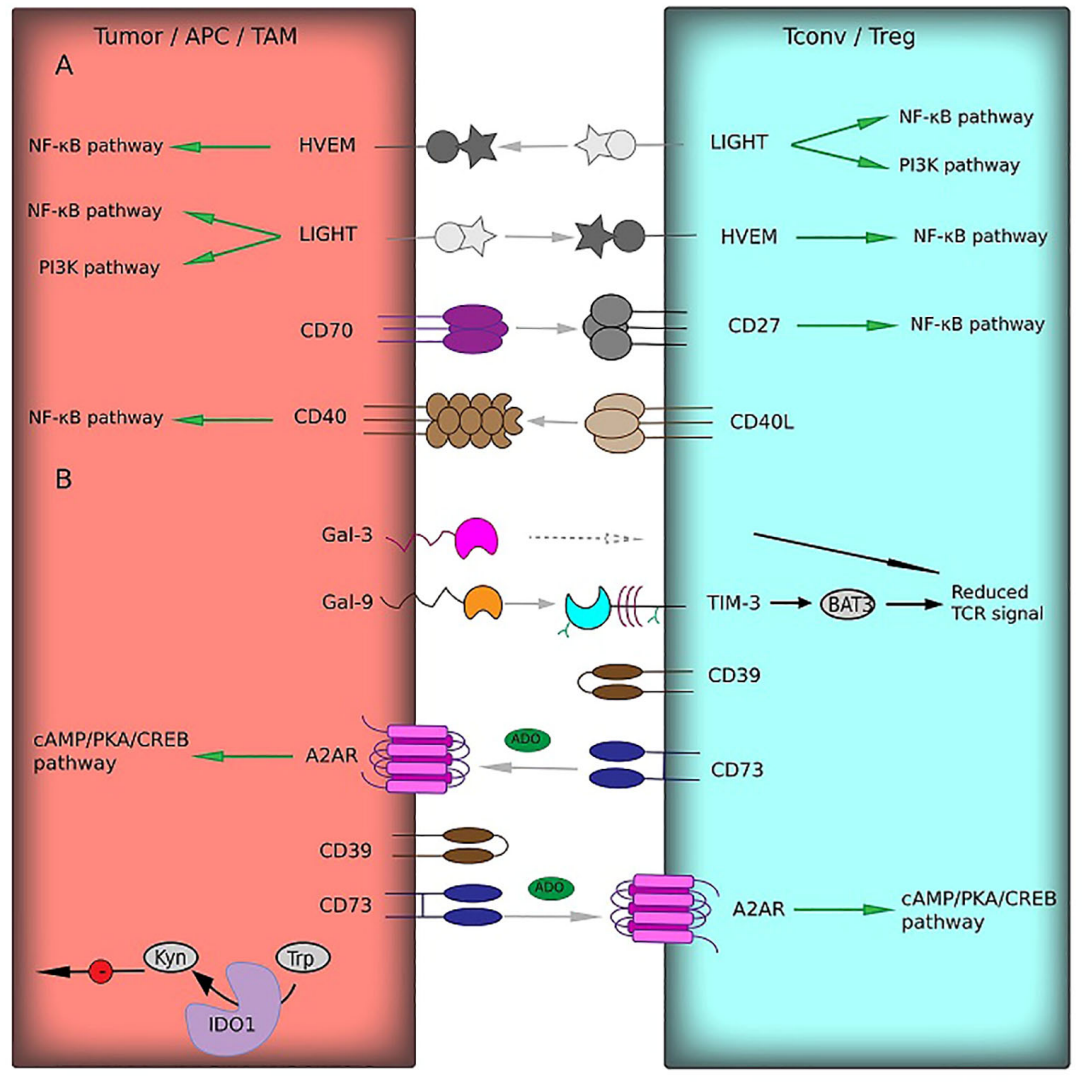
Receptor–ligand interactions (gray arrows) of immune checkpoints from the TNF-TNFR superfamily **(A)** and other protein families **(B)** between T cells or NK cells and tumor cells or TAM. In most cases, these interactions induce co-stimulatory signals (green arrows) predominantly via NF-κB pathway. Immune checkpoints from other families could influence TCR signaling (black arrows) or maintain functions of immunosuppressive cells (green arrows). Multiple negative effects of kynurenine on APC functions are shown by black arrows with red circles. BTLA and LAG-3, which interact with HVEM and Gal-3, respectively (gray dotted arrows), belong to IgSF and are presented in **Figure 1**.

LIGHT, also known as TNFSF14, is another TNF superfamily member and a ligand for HVEM. As mentioned above, there is a strong correlation between HVEM and LIGHT expression in glioma microenvironment. LIGHT expression directly correlates with the glioma grade (104). Moreover, LIGHT overexpression has been associated with a highly aggressive tumor phenotype (IDHwt and mesenchymal subtype) (104). In GBM patients, it correlates positively with poor survival. There is evidence that LIGHT promotes tumor growth in gliomas in a HVEM-dependent manner (105). The LIGHT/HVEM pathway, similarly to the canonical TNF/TNFR pathway, activates NF-κB and PI3K through the TNF receptor-associated factors, triggering survival signaling and transcription of the inflammatory genes (**Figure 2A**) (98). LIGHT was found to be co-expressed with PD-1/PD-L1, TIM-3, B7-H3, and other inhibitory immune checkpoints (104).

### 3.2 CD70 – CD27

CD70 is a well-studied TNF-like ligand that functions as a co-stimulatory molecule for T and B cells. CD70 is usually not expressed in a healthy tissue, except for peripheral blood leukocytes. It contributes to homeostatic signaling supporting lymphocyte survival in the absence of signals from TCR (106). CD70 levels are increased in several malignancies including 10% of primary LGGs and 35% of GBMs. GBMs and LGGs expressing CD70 have similar molecular characteristics and patient survival rates (107). Elevated CD70 has been linked to a poor prognosis in LGG patients with IDHwt. Most GBMs expressing CD70 have a mesenchymal phenotype, which negatively correlates with patients’ survival (107).

CD70 binds to the receptor CD27. CD27 is also a member of the TNFR family and is often presented on naïve and memory lymphocytes, NK cells, and mature DCs (108). As a co-stimulatory immune checkpoint, CD27 plays an essential role in survival and activation of T cells. CD70 expression stimulates tumor infiltration with immune cells, but it has no effect on CD27 expression. The possible explanation of this phenomenon lies in the CD70-dependent infiltration with macrophages instead of T cells (109) or the activation and generation of tumor Tregs triggered by CD27/CD70 (110). It is also possible that CD70 signaling can be mediated by an unknown inhibitory receptor on the T cell surface (107).

### 3.3 CD40 – CD40L

The CD40 glycoprotein and its ligand CD40L (CD154) are members of the TNF and TNFR superfamilies. CD40L is primarily expressed by the activated CD4^+^ T cells. CD40 expression is triggered by CD40L binding and is typical of B cells, macrophages, and DCs. Still, the roles of both receptor and ligand in tumor progression remain disputable. CD40 and its ligand were shown to be co-expressed on the surface of GBM cells (111). Interestingly, WHO grade III gliomas express higher CD40 and CD40L levels compared to GBMs. It was noted that the overexpression of both proteins could be associated with better overall and progression-free survival of GBM patients after tumor resection (112). However, high CD40 expression was detected in glioma biopsy samples and correlated with lesions and an increased vascularization (112). In another study, lower overall and progression-free survival rates were detected in LGG patients as well as patients with GBM expressing IDHwt and high levels of CD40. CD40 was upregulated in secondary gliomas as contrasted with primary gliomas. Werner and colleagues (113) did not find any correlation between CD40L expression and the overall survival rate; however, recent studies pointed out the negative correlation between CD40L levels and the disease outcome (114).

## 4 Other immune checkpoints/immune controlling molecules and mechanisms

### 4.1 TIM-3

T cell immunoglobulin domain and mucin domain protein 3 or TIM-3, also known as Hepatitis A virus cellular receptor 2 (HAVCR2), is a surface receptor found in most lymphocytes and cells of myeloid origin. TIM-3 promotes CD8^+^ exhaustion and apoptosis, reduces IL-2 and IFN-ɣ production (115). TIM-3 is one of the most upregulated co-inhibitory immune checkpoints in cancer, especially in glioma. There is a proven positive correlation between TIM-3 and the WHO grade, the mesenchymal phenotype, and a worse prognosis (116). TIM-3 promotes tumor progression by inducing the macrophage migration and tumor-promoting M2 polarization via the IL-6/NF-κB pathway (116). TIM-3 is co-expressed with PD-1, LAG-3 (117), VISTA, PSGL1, and Galectin-9 (Gal-9) (55).

Gal-9 is the member of the galectin protein family. Gal-9 expression is typical of glioma and depends on the WHO grade and TIM-3 levels (118). Gal-9/TIM-3 interaction has been shown to induce exhaustion and apoptosis of Th1, but not Th2 cells (**Figure 2B**). Gal-9 also binds to PD-1 and shields it from PD-L1, promoting the resistance of TIM-3-positive T cells to cell death (119). Gal-9 co-expression with PD-L1 and their co-localization in some GBM cases serve as further evidence for the link between TIM-3/Gal-9 and PD-1/PD-L1 pathways as supported by published data (118). Gal-9 also was shown to correlate with the expansion of M2 macrophages and MDSCs in GBM tissues (120, 121).

### 4.2 Adenosine pathway

A2AR, the immunosuppressive adenosine 2A receptor, is a member of the G protein-coupled receptor family. A2AR is expressed in the majority of immune cells, such as lymphocytes and cells of myeloid origin. This receptor tightly regulates adaptive immune responses via high affinity binding to adenosine. Their interaction triggers the cAMP/PKA/CREB pathway resulting in a reduced immune response (122). The adenosine/A2aR pathway was hijacked by tumor cells to evade the immune system. Currently, there is no evidence on the correlation between A2AR and a poor prognosis for GBM patients. Nevertheless, A2AR expression was reported to be a high-risk factor in the bioinformatics analysis of glioma samples (72). A2AR was upregulated in CD4^+^ and CD8^+^ glioma-infiltrating cells, its high levels being linked to the PD-1 and CD39/CD73 axis (72).

Although CD39 and CD73 are not fully recognized as immune checkpoints, they significantly contribute to tumorigenesis through ecto-5′-nucleotidase activity, which metabolize ATP to adenosine, and are commonly expressed on most B cells and monocytes and on some T cells. Under normal conditions, ATP is localized in the intracellular space, and its extracellular concentration grows during neuron release or in response to ischemia or hypoxia, which induces local inflammation (123). CD39 converts ATP to ADP and AMP, while CD73 converts AMP to adenosine, thereby switching the proinflammatory status of the microenvironment to the anti-inflammatory state (124). These events contribute to tumor growth, migration, and T cell function. CD73 expression was typically observed in tumor macrophages and Tregs, which directly inhibit the cytokine release and cytotoxic functions of CD8^+^ T cells (125). Importantly, according to the recent studies, CD39 and CD73 are usually co-expressed in tumor cells and their simultaneous action causes adenosine-dependent pro-tumor immune suppression (126). Downregulation of both CD39 and CD73 in TME correlated with a better prognosis for patients (127).

### 4.3 IDO1

Indoleamine-2,3-dioxygenase (IDO1) is an immune checkpoint secreted molecule involved in tryptophane metabolism. IDO1 is widely expressed in various healthy tissues, including lung and gastrointestinal tract tissues, placenta, and immune cells. IDO1 can suppress T cell function and help to maintain the immune privileged status of some tissues like placenta and fetus (128). The immunosuppressive IDO1 pathway is involved in converting tryptophan into kynurenine. Tryptophan starvation activates general control nonderepressible 2 (GCN2), a serine/threonine kinase that phosphorylates eukaryotic initiation factor 2α kinase (eIF2α). These changes lead to decreased transcriptional activity and reduced fatty acid production (129). Kynurenine, in turn, activates the aryl hydrocarbon receptor and induces DC immune tolerance (129). IDO1 upregulation is commonly observed in cancer. In glioma, IDO1 levels positively correlate with the WHO tumor grade, IDHwt status, the mesenchymal subtype, and Treg expansion (130). IDO1 expression depends on IFN-ɣ release creating a trap for tumor-infiltrating effector and cytotoxic T cells (131). IDO1 is co-expressed with PD-L1, PD-L2, PD-1, CTLA-4, CD39, BTLA, and LAG-3. Taken together, these characteristics of IDO1 indicate a strong correlation between IDO1 expression and lower overall survival of GBM patients (130).

## 5 Glioma immunotherapy: limitations and perspectives

As described above, immune checkpoints regulate immune responses, creating immunosuppressive TME and maintaining glioma development. Nevertheless, blocking co-inhibitory immune checkpoints can restore antitumor immune activity of the effector cells. PD-1/PD-L1 blockers demonstrated clinical benefits in various neoplasms (132).

As PD-1/PD-L1 monotherapy has not been successful in case of glioma, the combinations of blockers are created with anti-PD-1 as the first component (**Table 1**). Co-expression of PD-1 and CTLA-4 has been demonstrated for many tumors, which formed the basis for combination target therapy. Dual PD-1 and CTLA-4 inhibition demonstrated high efficiency for several tumors (133). Co-expression profiles of PD-1 and CTLA-4 in glioblastoma gave rise to the clinical trials of the corresponding blockers. The antibodies were proven to be safe, but didn’t improve the survival of patients (**Table 1**). As a result, an intensive development and testing of new blockers of the alternative immune checkpoints is currently underway. The novel blockers of LAG-3, TIM3, IDO1 and TIGIT are emerging and under testing in clinical trials (134).

Brain tumor therapy faces several challenges which the scientific community is focused on. Due to the high proportion of immunosuppressive macrophages from the tumor mass, antibodies targeting TAM receptors (such as CSF-1R) or the chemokine recruitment system are under development (135). To overcome the problem of BBB crossing, local chemotherapy is preferable to systemic therapy (136). TMZ and corticosteroids was shown to act depressively on the weakened immune system of the patient (137). Neoadjuvant therapy could help protect the effector cells against the negative side effects of chemotherapy. Several groups indicated greater effectiveness of neoadjuvant therapy compared to adjuvant one. Therapies with neoadjuvant nivolumab (138) or pembrolizumab (139) are being actively developed, showing promising results.

The expression of an alternative immune checkpoints and tumor heterogeneity in expression profiles are proposed to be treated with combined methods or multivalent inhibitors (140). The problem of tumor heterogeneity is increasingly proposed to be solved by personalized treatment based on the individual transcriptomic, metabolomic, and proteomic profiles (141). It will allow selecting an individual combination of targeted therapies for each patient. In addition, more and more alternative checkpoints are involved in targeted therapy. Mutually exclusive expression of PD-1 with B7-H3 and B7-H4 make them appealing markers and targets for combined therapy with anti-PD-1. Evaluation of this molecules in diagnostics may help to identify and better understand biology of cells non-sensitive to anti-PD-1 treatment. T cells with chimeric antigen receptors (CAR T) targeting B7-H3 in GBM are currently in phase I trials (NCT05241392, NCT04385173, NCT04077866, NCT05366179) (142). The antibodies to CD39 (IPH5201, NCT05742607) and CD73 (IPH5301, NCT05143970) are in phase I trials and may also be feasible for GBM (143). The clinical relevance of other immune checkpoints in GBM is still disputable.

## 6 Conclusion

GBM is the most aggressive glioma subtype with high resistance to therapy and an extremely low median patient survival. Low susceptibility to treatment is caused by the formation of TME with a remarkably complex molecular and cellular network. Along with tumor cells, TME comprises stromal cells, epithelial cells, and, importantly, tumor-infiltrating immune cells that fail to eliminate the tumor. Glioma cells, TILs, and TAMs express a variety of inhibiting molecules that contribute to the tumor immune escape (**Table 2**). It is interesting that immunomodulatory proteins are commonly present on the tumor parenchyma surface and in TME cells. Apparently, these molecules activate the immunosuppressive subtypes of immune cells mostly due to their preferential infiltration or/and generation. Nevertheless, the tumor origin and the molecular expression profile of the surrounding non-immune cells should also be taken into account, as some protein markers could serve as predictors of both good and bad disease outcomes, depending on the tumor type (48, 66, 67). Thus, most of the expressed immune checkpoints on GBM cells and their environment are associated with a poor prognosis. LGG is characterized by a smaller range of expressed immune checkpoints compared to HGG. The majority of detected proteins in LGG such as CTLA-4, B7-H3, LAG3, CD155, CD70 and CD40 were associated with worse outcomes of the disease as in HGG. The only exception established is B7-H7 or HHLA2, whose expression is more typical for LGG and is associated with a better prognosis.

**Table 2:** Immune checkpoint molecules involved in glioma development. Information not included in the main text is provided as references.

Molecule	Protein family	Cells expressing	Pathways	Effects	Co-expression profiles	Association and prognosis
PD-1	IgSF, CD28 family	Activated T cells and B cells	Recruiting SHP-2 followed by dephosphorylation of signaling molecules, sequestering CD80 away from CD28 by PD-L1	Apoptosis and exhaustion of Tconv, generation Tregs and TAMs	Co-expressed with majority of inhibiting immune checkpoints	GBM-induced Tregs, worse disease outcome
PD-L1	IgSF, B7 family	Tregs, activated Tconv, macrophages, tumors				
PD-L2	IgSF, B7 family	DCs, macrophages, tumors			PD-1, PD-L1	IDHwt mesenchymal GBM, conferred poor prognosis
CTLA-4	IgSF, CD28 family	Tregs, activated Tconv	Inhibits AKT phosphorylation, blocks CD80 and CD86 interaction with CD28	Lower Tconv activation, Treg expansion	PD-1, CD40, ICOS and TIGIT	IDHwt status and mesenchymal cell type of HGG, higher Treg and TAM infiltration, lower survival probabilities
ICOS	IgSF, CD28 family	Activated T cells	Activation of NF-κB signaling	Activation of both Tconv and Tregs	PD1, PD-L1, PD-L2, CTLA-4, ICOSLG and IDO1	IDH wild type, and mesenchymal subtype of gliomas with higher grade Overexpression of ICOS in TME and lower ICOS expression in blood plasma of patients was associated with lower survival
ICOSLG	IgSF, B7 family	APC, somatic cells, monocytes			ICOS, PD-1, PD-L1, CTLA-4, IDO1, TIM-3 (144)	GBM stem cells, mesenchymal phenotype and IL-10-producing T cells
B7-H3	IgSF, B7 family	Endothelial cells, fibroblasts, osteoclasts, stromal cells, APC, NK, activated T cells, tumor	Activation of JAK2/STAT3 survival pathway	Tumor immune escape, survival and growth	Low correlation with B7-H4	IDHwt and higher grade glioma, associated with lower survival
B7-H4	IgSF, B7 family	APC, tumor	Activation of JAK/STAT pathway in IL-6-dependent manner	Tumor immune escape	Low correlation with B7-H3	Elevated number of TAMs, positively correlate with tumor grade and poor prognosis
VISTA	IgSF, B7 family	Myeloid cells, TILs	Inhibits NF-κB signaling pathway	Reduced proliferation and cytokine production by Tconv	B7-H3, PD-1, PD-L1, LAG-3, TIM-3, PSGL1	Correlates with WHO glioma grade and poor prognosis
VSIG3	IgSF	Tumors			VISTA, no other co-expression data for GBM	
PSGL1	Selectins	Myeloid cells, activated T cells			VISTA, no other co-expression data for GBM	No data for glioma
B7-H6	IgSF, B7 family	Tumors	Activates PI3K/Akt, ERK/MAPK and c-Myc/RNMT signaling pathways	Control of a variety of biological processes such as proliferation, migration, invasion, survival etс	PD-L1 (61)	Positively correlates with tumor aggressiveness and poor prognosis
B7-H7	IgSF, B7 family	Endothelial cells, tumors	Promotes AKT phosphorylation	Enhanced T cell proliferation and cytokine production	No data for glioma	Correlated with lower grade glioma and prolonged overall survival
HVEM	TNFRSF	Epithelial cells, mesenchymal cells, majority of immune cells	Activates NF-κB pathway	Stimulation and proliferation of HVEM-expressing cells	TIM-3, PD-1, PD-L1, CTLA-4, LAG-3 and VISTA	Indicates higher-grade glioma with increased immune and stromal cells in TME, predicts poor outcome
BTLA	IgSF, CD28 family	Majority of immune cells	Recruits SHP-1 or SHP-2 followed by dephosphorylation of signaling molecules, shielding HVEM molecules from LIGHT	Inhibition of function of BTLA-expressing cells	HVEM, LAG-3, TIM-3 (145)	No data for glioma
LIGHT	TNFSF	T cells, macrophages	Activates NF-κB and PI3K through TNF receptor	Stimulate survival and proliferation while interacts with HVEM	HVEM, PD-1, PD-L1, TIM-3, B7-H3	IDHwt and mesenchymal glioma subtype
LAG-3	IgSF	Activated T cells, myeloid cells	Interrupts TCR signaling by MHCII binding (146)	Triggers T cell exhaustion	CTLA-4, PD-1 and TIM-3	Associated with PD-L1^+^ IDHwt glioma cells and PD-1^+^ TILs, considered as risk factor in GBM
Gal-3	Galectins	Macrophages, tumors	Triggers surface LAG-3 and transmits inhibiting signal in T cells		No data for glioma	Correlates with WHO grade of gliomas
FGL1	Fibrinogen family	Soluble protein produced by hepatocytes in liver (147)				No data for glioma
CD70	TNFSF	Malignancies, less often – activated T cells and NK	Activates NF-κB pathway	Enhances activation of TAMs and T cells including Tregs	No data for glioma	IDHwt mesenchymal GBM and lower survival
CD27	TNFRSF	Naïve and memory lymphocytes, NK, mature DCs				Not widely represented in glioma TME
CD40	TNFRSF	B cells, macrophages and DCs	Activates NF-κB pathway	Enhanced adhesion and cytokine production	CD40L, no other co-expression data for GBM	Data is controversial, CD40 could be both positive (111) and negative factor (112, 113).
CD40L	TNFSF	Activated T cells			CD40, no other co-expression data for GBM	Data is controversial, CD40L could show good prognosis (111), bad prognosis (114), or no correlation with overall survival (113)
CD155	IgSF	Malignant cells, rarely epithelial or endothelial cells	Shielding CD155 and CD112 from CD226 by TIGIT, suppressing PI3K, MAPK, and NF-κB pathways (148)	Depletion of T and NK cells and less cytokine production	PD-L1, PD-1	Related to increased metastasis of GBM, promotes TIGIT^+^ immune cell infiltration
CD112	IgSF	Macrophages, monocytes, some healthy tissues			CD155	No data for glioma, probably the same role as for CD155
TIGIT	IgSF, CD28 family	Memory and activated T cells, Tregs, NK cells, NKT cells			PD-L1, PD-1, CD47 (149), CD226 (82)	Associated with poor overall survival
CD200	IgSF	Majority of lymphoid cells, stromal cells, tumors	Inhibits Ras and downstream ERK activation (150)	Induction of M2-macrophages and MDSC	No data for glioma	Increased accumulation of MDSC in glioma TME, no association with prognosis
CD48	IgSF	APC, NK, tumors	Recruits SHP-1 or SHP-2 as an inhibitor or SAP as an activator	Induce T cell activation and inhibit NK cell functions	TIGIT, ICOS, TIM-3	TAM and TILs infiltration, IDHwt status of mesenchymal subtype glioma and worse outcome
TIM-3	Transmembrane immunoglobulin and munin domain (TIM) proteins	Majority of immune cells, especially T cells	Releases BAT3 which activates tyrosine kinase LCK and inhibits TCR signaling, activates IL-6/NF-κB pathway in macrophages	Multiple immune effects such as apoptosis of Th1 cells, CD8^+^ exhaustion, apoptosis, reduced cytokine production	PD-1, LAG-3, VISTA, PSGL1 and Gal-9.	Correlates with WHO grade, mesenchymal phenotype and worse prognosis for patients
Gal-9	Galectins	Tumors, bone marrow and lymphoid tissues			TIM-3, PD-1, PD-L1	Correlates with M2 macrophage and MDSC expansion
A2AR	G protein-coupled receptor (GPCR) family	Majority of immune cells	Activates cAMP/PKA/CREB pathway during binding adenosine	Reduced adaptive immune responses	PD-1, CD39, CD73	High-risk factor in glioma
CD39	Ectonucleotidases	B cells, monocytes, T cells (especially Tregs)	Converses ATP to ADP and AMP		CD73, IDO1	Both are associated with worse prognosis
CD73	Ectonucleotidases	B cells, monocytes, T cells (especially Tregs)	Converses AMP to adenosine		CD39, CD155 (151), A2AR (152)	
IDO1	Oxidoreductases	Immune privileged tissues and malignancies	Breaking down tryptophan into kynurenine	Makes DCs inactive	PD-L1, PD-L2, PD-1, CTLA-4, CD39, BTLA, LAG-3	Positively correlates with WHO tumor grade, IDHwt, mesenchymal subtype and Treg expansion
CD47	IgSF	T cells, NK, DCs, tumors	Recruits SHP-1 and SHP-2 in APC or activates PI3K/Akt pathway in CD47^+^ cells	Reduced phagocytosis of APC and immune escape of tumor	TIGIT	Associated with glioma stem-like cells and predicts worse prognosis for patients
CD277	IgSF, butyrophilin family	T cells, B cells, NK, DCs, tumors	Activation of IL-6/JAK/STAT3 pathway	Increased infiltration of immune cells and CD8^+^ T cell exhaustion	TIM-3	IDHwt glioblastoma with high Treg infiltration

Since the majority of the regulatory molecules described above are co-expressed, they seem to be involved in the same complex regulatory cascade, or even a suppressive signaling network. The activation of this “network” triggers multiple immunosuppressive effects causing a gradual amplification of inhibitory signals. This affects a wide range of cell types: from healthy brain tissues and glioma cells to cytotoxic lymphocytes in the TME and the peripheral blood. Moreover, most molecules were associated with the end-stage glioma, mesenchymal glioblastoma with wild-type IDH1. However, the mechanisms of immune checkpoints synergy underlying glioma development are still far from being fully understood. Most studies reported PD-1/PD-L1 and CTLA-4 signaling to be a key to further suppression of the immune system (153). Nevertheless, anti-CTLA-4 and anti-PD-1 therapy appear not to be as effective as was anticipated (35, 154). This fact points to the possible existence of another “switch” molecule that initiates the disease terminal stage. The promising candidates for glioma therapy include some interleukins, such as IL-6 (155), IL-17 (156) or IL-20 (157), or chemokines (158).

## Glossary

APC: Antigen-presenting cells
CNS: Central Nervous System
DC: Dendritic cells
GBM: Glioblastoma multiforme
HGG: High-grade gliomas
IgSF: Immunoglobulin superfamily
ITSM: Immunoreceptor tyrosine-based switch motif
LGG: Low-grade gliomas
MDSC: Myeloid-derived suppressor cells
mOS: Median overall survival
NK: Natural killer cell
RT: Radiation Therapy
SHP-2: Src homology region 2 domain-containing phosphatase-2
TAM: Tumor-infiltrating macrophages
Tconv: Conventional T cells
TCR: T-cell receptor
TIL: Tumor-infiltrating lymphocytes
TME: Tumor microenvironment
TMZ: Temozolomide
TNF: Tumor necrosis factor
TNFR: Tumor necrosis factor receptor
Treg: Regulatory T cell
